# Effect of Non-surgical Periodontal Therapy on Serum and Salivary Concentrations of Visfatin in Patients with Chronic Periodontitis

**DOI:** 10.15171/joddd.2015.003

**Published:** 2015-03-04

**Authors:** Nader Abolfazli, Sahar Jabali, Fariba Saleh Saber, Zohreh Babaloo, Adileh Shirmohammadi

**Affiliations:** ^1^Associate Professor, Department of Periodontics, Faculty of Dentistry, Tabriz University of Medical Sciences, Tabriz, Iran; ^2^Assistant Professor, Department of Periodontics, Faculty of Dentistry, Urmia University of Medical Sciences, Urmia, Iran; ^3^Associate Professor, Department of Prostodontics, Faculty of Dentistry, Tabriz University of Medical Sciences, Tabriz, Iran; ^4^Associate Professor, Department of Immunology, Faculty of Medicine, Tabriz University of Medical Sciences, Tabriz, Iran

**Keywords:** Chronic periodontitis, serum, visfatin

## Abstract

***Background and aims.*** Visfatin, mainly secreted by visceral adipose tissue, especially by macrophages, plays an important role in regulating the defense and immune functions, and functions as a growth factor, a cytokine, an enzyme and more importantly as a proinflammatory mediator. The aim of the present study was to evaluate the effect of non-surgical periodontal treatment on serum and salivary levels of visfatin in patients with generalized moderate-to-severe chronic periodontitis.

***Materials and methods.*** Eighteen patients with generalized moderate-to-severe chronic periodontitis were selected based on periodontal parameters of gingival index (GI), probing pocket depth (PPD), clinical attachment level (CAL) and radiographic parameters. Serum and salivary samples were collected at baseline and one month following non-surgical periodontal therapy (scaling and root planing ([SRP]). Visfatin levels were measured using an ELISA kit. Data were analyzed by SPSS 15, using paired t-test and Pearson's correlation coefficient.

***Results.*** Mean salivary and serum levels of visfatin significantly decreased after non-surgical periodontal treatment (P<0.05). Changes in salivary visfatin levels were more prominent.

***Conclusion.*** According to the findings of this study it seems that there is a direct relationship between periodontal tissue inflammation and disease activity with salivary and serum visfatin levels.

## Introduction

Periodontal disease is a chronic inflammatory condition and is initiated by accumulation of bacterial plaque in the gingival sulcus, which induces an inflammatory response.^1^ Apart from direct bacterial effect on periodontal tissues, damage to periodontium can be induced by indirect methods, too. Destruction of the protective components of the periodontium by bacterial virulence mechanisms results in the exposure of cells and underlying tissues to bacterial agents and initiation of a number of destructive processes. As a result, cellular agents, such as monocytes, lymphocytes and fibroblasts are stimulated by bacterial constituents, including lipopolysaccharides (LPS), resulting in the secretion of cytokines and proinflammatory mediators such as IL-6, IL-1, PGE-2, TNF-α and IL-8. These cytokines have the capacity to function alone or in concert with each other to induce inflammatory responses and catabolic processes, including bone loss and degradation of collagen by matrix metalloproteinases.^2^ Although microorganisms are etiologic agents for inflammatory lesions, proinflammatory mediators, too, have a major role in connective tissue loss and destruction of the supporting alveolar bone.^3^ The adipose tissue is an endocrine organ which secrets various soluble factors such as adiponectin, liptin and resistin, collectively referred to as adipocytokines or adipokines. Evidence has shown that adipokines released from the adipose tissue are involved in a wide range of physiologic and pathologic processes, including immunity and inflammation.^4^ Fukuhara et al5 identified a new adipokine in 2005, referred to as visfatin (visceral fat adipokine).

Visfatin is a multi-potential mediator which functions as a growth factor, a cytokine, an enzyme with a role in energy metabolism and a proinflammatory mediator. It is a 52-KDa protein, which was initially introduced as a factor increasing pre B-cell colony (PBEF), which is a growth factor released from lymphocytes and has a role in the maturation of B lymphocytes.^6^ Visfatin is also known as nicotineamide phosphoribosyl transferase (NAMPT), which is an enzyme inhibiting the biosynthesis of nicotineamide-adenine dinucleotide (NAD).^7^ Visfatin is mainly secreted by visceral adipose tissue, especially by macrophages,^8,9^ and can also be secreted by various tissues and cells, including lymphocytes, muscle cells, XXXisfatinXXXes, dendritic cells and bone marrow cells.^7,10^ Presence of visfatin in a wide range of white blood cells and macrophages bonded to tissues indicates that it has an important role in the regulation of defense and immune response.^10^ It can be considered an inflammatory adipokine since it can be found in inflammatory cells and in various inflammatory conditions. Studies have shown that expression of visfatin increases in acute and chronic inflammatory conditions, including rheumatoid arthritis, sepsis,^12^acute pulmonary conditions,^13^ psoariasis,^14^ and type II diabetes mellitus.^15^ High serum levels of visfatin are considered a risk factor for cardiovascular diseases^16,17^ and an increase in the expression of visfatin by macrophages in unstable atherosclerotic lesions indicates the possible role of visfatin in destabilization of the atherosclerotic plaque.^18^

An increase in cytokine levels in periodontal diseases might have an important role in predicting the high risk of these patients for systemic conditions, such as atherosclerosis, cardiovascular diseases, diabetes, rheumatoid arthritis and premature birth.^19^ Since an increase in proinflammatory cytokines, such as TNF-α, IL-6 and IL-1β, results in a significant increase in the expression of XXXisfatin,^20^ a high level of visfatin in periodontal disease might exacerbate or induce such diseases; in this context, since treatment of periodontal diseases decreases the inflammatory load, it might decrease the risk of such diseases not only in patients at a high risk for these systemic conditions but also in healthy individuals.^21^ Visfatin might be considered a possible marker in the inflammatory activity of periodontal diseases.^22^ However, only a limited number of studies have evaluated the role of visfatin in periodontal diseases and the results have shown a direct relationship between visfatin levels and the progress of periodontal disease. In 2011 Pradeep et al evaluated the relationship between the serum and gingival crevicular fluid (GCF) concentrations of visfatin and periodontal diseases and determined its concentrations in 3 groups of healthy individuals and patients with gingivitis and periodontitis. They concluded that concentrations of visfatin in the serum and the GCF progressively increase from health to gingivitis to periodontitis and with an increase in the severity of periodontal diseases.^23^ In another study in 2011, Pradeep et al evaluated the relationship between serum and GCF concentrations of visfatin in 10 healthy subjects, 10 patients with chronic periodontitis with proper control of type II diabetes mellitus and 10 patients with chronic periodontitis without diabetes, concluding that concentrations of visfatin were higher in patients with periodontal disease and type II diabetes mellitus than those in patients with periodontal disease but without type II diabetes and healthy individuals.^22^ Ranghavendra et al evaluated the effect of non-surgical periodontal treatment on serum and GCF levels of visfatin and reported that serum and GCF concentrations of visfatin decrease after treatment and reach the levels during periodontal health.^21^

Since SRP is considered the gold standard of non-surgical periodontal treatment^24^ and since there are only a limited number of studies on the effect of non-surgical periodontal treatment on visfatin levels, and since to the best of our knowledge no studies so far have evaluated the salivary levels of visfatin, the present study was undertaken to evaluate the serum and salivary concentrations of patients with generalized moderate-to-severe chronic periodontitis before and after non-surgical periodontal treatment so that the results would be used to confirm or refute the results of previous studies.

## Materials and Methods

The study group consisted of 18 patients with generalized moderate-to-severe chronic periodontitis, who had referred to the Department of Periodontics at Tabriz University of Medical Sciences Faculty of Dentistry between April 2012 and February 2013. Inclusion criteria consisted of a BMI (BMI = weight/height^2^) of 18-24.9 kg/m^2^, presence of at least 20 teeth in the oral cavity, GI>1, a diagnosis of generalized moderate-to-severe chronic periodontitis with at least 30% of the areas undergoing clinical attachment loss of at least 3 mm and a probing depth of ≥5 mm and radiographic evidence of bone loss. Exclusion criteria consisted of patients with systemic conditions, hypertension, aggressive periodontitis, pregnancy or breastfeeding, a history of taking antihyperlipidemic agents or systemic antibiotics (during the previous 6 months), continuous use of NSAIDs, a history of smoking, and a history of SRP during the previous 6 months.

The protocol of the study was approved by the Ethics Committee of Tabriz University of Medical Sciences. At first all the subjects were examined and if they met the inclusion criteria, they received an explanation about the study protocol and informed written consent was obtained. Clinical parameters of GI (gingival index), CAL (clinical attachment level), and PPD (probing pocket depth) were used to confirm a diagnosis of periodontitis. The measurements were performed at baseline and one month postoperatively by a masked calibrated examiner (NA).

PPD is the distance from the gingival sulcus to the deepest point of probe penetration into the gingival sulcus using the standard technique. PPD was registered at four mesiobuccal, midbuccal, distobuccal, and midligual/midpalatal areas in the periodontal chart.

CAL is the distance from the CEJ to the most distal point the probe penetrates into the sulcus or the pocket using the standard technique.^26^

GI is used to evaluate the extent and severity of gingival inflammation.^27^ Periapical radiographs with the use of long cone technique were used to evaluate the presence or absence of bone loss in order to make a distinction between individuals with chronic periodontitis and those without it.

Sampling for serum and salivary levels of visfatin was carried out in the beginning. To this end, at first the patients received oral hygiene instructions (brushing with the modified Bass technique and use of dental floss). The patients were fasting for 12 hours after primary oral hygiene measures when sampling was carried out; 3 mL of salivary sample and 5 mL of blood were taken from 8 to 10 in the morning. SRP was carried out using hand and ultrasonic instruments and repeated during follow-up sessions if needed for 4 weeks. All the clinical procedures were carried out by a postgraduate student (S.J). O’Leary’s plaque index was recorded before and after treatment in an attempt to achieve and maintain the index at values under 20% by instructing hand plaque control techniques.^28^ Salivary and serum samples were also taken one month after scaling and immediately sent to the laboratory for the evaluation of visfatin biomarker.

### Collection of Salivary and Serum Samples

Passive drooling method was used to collect unstimulated whole saliva samples, during which the participants drooled down a straw and the samples were collected in a plastic vial. The salivary samples were centrifuged at 3000 rpm for 10 minutes. Blood samples were taken from the forearm veins using 5-mL syringes. The blood samples were coagulated at room temperature and after one hour were centrifuged at 3000 rpm for 10 minutes to separate the blood serum. The samples were stored at -70ºC until they were evaluated.

### Visfatin Assay

Visfatin concentrations of the samples were assayed by double-antibody sandwich enzyme-linked immunosorbent assay (ELISA) kit (Glory Science Co., Ltd, USA). The minimum assay sensitivity of the kit was <0.25 ng/mL.

Analysis of the samples was carried out in the Department of Immunology at Applied Drug Research Center, Tabriz University of Medical Sciences, Tabriz, Iran. The kit was conditioned for at least 30 minutes before the assay at room temperature (18-28°C).

In favorable circumstances, the double-well assay is recommended. The blank well was assumed as zero and only chromogen solutions A and B and stop solution were incorporated. For the standard 50-µ standard well, 50 µL of streptavidin-HRP was added; other protocols were the same as those with the test wells. For each test well, a 40-µL sample was added to the well which was pre-coated with human visfatin monoclonal antibody; subsequently, 10 µL of visfatin-antibody labeled with biotin and 50 µL of streptavidin-HRP were added to create an immune complex, which was gently shaken and incubated for 60 minutes at 37°C. The wells were washed 5 times with 300 µL of wash solution to eliminate the unreacted enzyme; 50 µL of chromogen solution A and 50 µL of chromogen solution B were added to each well and incubated for 10 minutes at 37°C in a dark environment. A total of 50 µL of stop solution was added into each well to stop the reaction (immediately the blue color changed into yellow). For final measurements the blank well was assumed as zero and the absorbance of the substrate color reaction was read on an ELISA plate reader (Awareness, USA) under 450-nm wavelength, carried out within 10 minutes after incorporating the stop solution. The visfatin concentration in each well was reported as ng/mL.

### Statistical Analysis

Data was analyzed using SPSS 15 statistical software. At first data was analyzed for normal distribution; paired samples t-test was used when data was distributed normally. Non-parametric Wilcoxon’s test was used when data was not distributed normally. Statistical significance was defined at P<0.05.

## Results

**Figure 1.**
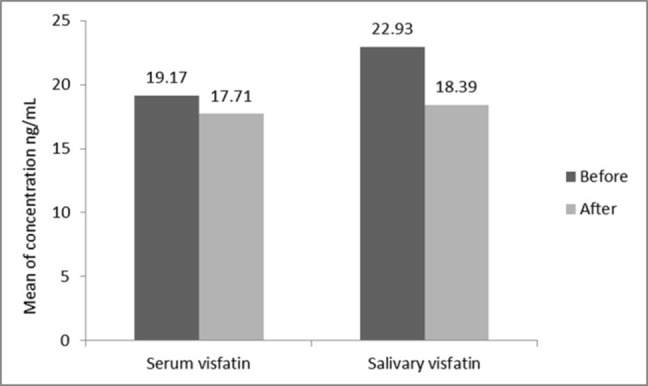
Comparison of salivary and serum levels of visfatin before and after treatment.

The descriptive statistics of the subjects are presented in Table 1. Of 18 subjects in the present study, 38.9% were male and 61.1% were female. Table 2 presents the results of paired t-test in relation to the comparison of salivary and serum levels of visfatin before and after treatment. The results showed a significant decrease in serum levels of visfatin after treatment (P<0.05). In addition, salivary levels of visfatin significantly decreased after treatment (P<0.05) (Figure 1).

**Table 1:** Descriptive statics (mean ± SD) of the study population

Group	Before treatment	After treatment	Age (years)	41.16±7.59	41.16±7.59
BMI (kg/m^2^)	24.35±0.63	24.35±0.63

**Table 2:** Results of paired t-test in relation to the comparison of mean salivary and serum levels of visfatin before and after treatment

	Mean ± SD				
Visfatin concentration (ng/mL)	Before treatment	After treatment	T-value	Range of freedom	P-value
Serum	19.17±4.24	17.71±4.71	2.439	16	0.027^*^
Salivary	22.93±9.73	18.39±8.61	2.22	17	0.043^*^
^*^Statistically significant at P < 0.05					

**Figure 2.**
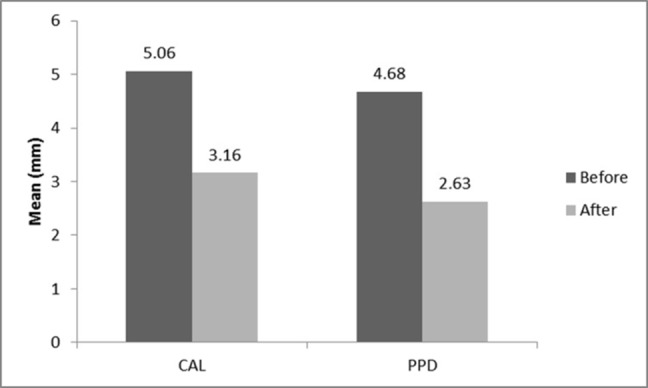
Comparison of clinical attachment levels (CAL) and probing pocket depths (PPD) before and after treatment.

The results of paired t-test in relation to the comparison of clinical parameters before and after treatment are presented in Table 3. Paired t-test showed that CAL, PPD, GI and PI exhibited decreases of 1.9±0.54 mm, 2.05±0.24 mm, 1.15±0.38 mm and 47.14±17.86%, respectively, after treatment, which were statistically significant (P<0.001; Figure 2).

**Table 3:** The results of paired t-test in relation to the comparison of mean salivary and serum levels of visfatin before and after treatment

Periodontal variables	Mean ± SD		T-value	Range of freedom	P-value
	Before treatment	After treatment			
CAL (mm)	5.0671±0.54	3.15±0.45	15.045	17	0.000^*^
PPD (mm)	4.67±0.51	2.63±0.45	36.637	17	0.000^*^
GI	1.85±0.27	0.71±0.33	12.732	17	0.000^*^
PI (%)	62.76±19.46	15.61±4.75	11.198	17	0.000^*^
^*^Statistically significant at P <0.05					

Pearson’s correlation coefficient was used to evaluate the relationship between serum and salivary levels of visfatin before and after treatment (Table 4). The test did not show any correlation between salivary and serum levels of visfatin. In addition, there was no correlation between salivary and serum levels of visfatin after treatment.

**Table 4:** Pearson’s correlation test for the salivary and serum levels of visfatin

Visfatin	Serum	
	Before treatment		After treatment	
	Correlation coefficient	P-value	Correlation coefficient	P-value
Salivary	Before treatment	-0.168	0.520		
	After treatment			-0.090	0.721

## Discussion

The aim of this study was to evaluate the serum and salivary level changes of visfatin following non-surgical periodontal therapy (SRP) in patients with generalized moderate-to-severe chronic periodontitis. The results of the preset study showed a significant decrease in salivary and serum levels of visfatin following non-surgical periodontal treatment. However, no significant relationship was observed between serum and salivary levels of visfatin either before or after treatment.

It has been suggested that visfatin has more potent destructive and proinflammatory properties and has a key role in the persistence of inflammation through inhibition of apoptosis and neutrophils.^39^ Visfatin has insulin-mimetic properties; it induces phosphorylation of substructures of 1 and 2 of insulin receptors in human osteoblasts, increases transfer of glucose, induces proliferation and production of Type I collagen, and induces angiogenesis.^40,41^ Visfatin levels increase in chondrocytes in response to IL-1β and acts in an autocrine and paracrine manner in the synthesis of PGE-2.^42^

Visfatin can be considered a possible marker in the inflammatory activity of periodontal disease;^22^ however, very few studies have evaluated the effect of periodontal treatment on visfatin levels. Recently, Raghavendra et al evaluated the effect of non-surgical periodontal treatment on serum and GCF levels of visfatin after 8 weeks and reported a decrease in serum and GCF levels of visfatin after treatment.^21^ The results of the present study were consistent with those of that study and a significant decrease in serum and salivary levels of visfatin was observed after improvements were made in the status of periodontal tissues and modification of periodontal variables.

An important difference between the present study and other studies was a difference in one of the samples, i.e. salivary samples were used instead of GCF samples, which facilitated and accelerated sampling procedures. In addition, in the present study the effect of treatment was evaluated after one month, which might explain a lower decrease in serum levels of visfatin in comparison to the study carried out by Raghavendra et al because visfatin level changes were evaluated at a shorter postoperative interval in the present study.

Since studies have shown that expression of visfatin increases in acute and chronic inflammatory conditions such as rheumatoid arthritis,^11^ acute pulmonary injury,^13^ and type II diabetes mellitus,^15^ a decrease in visfatin levels due to periodontal treatment might result in a decrease in the risk of diseases related to this factor. In addition, visfatin can act as a target to verify the potential of treatment in periodontal diseases.

In the present study, salivary and serum samples were used; however, in previous studies visfatin levels of serum and GCF have been evaluated. GCF is in close contact with periodontal tissues; therefore, it reflects the status of this tissue more properly. However, it is difficult to collect GCF samples.^30^ In contrast, there are copious amounts of saliva and it is easy to collect salivary samples with easy and non-invasive techniques, without a need for special skills. The patients can easily tolerate such procedures. On the other hand, saliva contains enzymes similar to those of the GCF.^31,32^ Saliva contains biomarkers specific for the unique physiologic aspects of periodontitis and quantitative changes of these markers might help diagnose such conditions. Evaluation of salivary levels of visfatin is easy and non-invasive and it is possible that it will become an acceptable alternative for the evaluation of inflammatory conditions associated with this biomarker.^32^Some recent studies have shown the presence of adipokines in saliva. Toda et al reported a relationship between salivary adiponectin and an increased risk of non-insulin-dependent diabetes mellitus and cardiovascular diseases.^33^ Micro et al introduced salivary leptin as a possible diagnostic marker for salivary gland tumors.^34^ In a study by Mamali et al, visfatin was identified in saliva but no significant relationship was found with its serum levels.^35^ It seems protein/polypeptide hormones cannot be identified in the saliva unless they are secreted by the salivary glands themselves. In other words, it is not possible for plasma protein hormones to passively diffuse into the saliva due to their large molecular size. Therefore, identification of these markers in the saliva can be considered a sign of plasma leakage from the oral cavity lesions, although some other mechanisms, such as active transport from the salivary glands and primary secretion, too, might be involved.^36^ In the present study, salivary visfatin levels were higher than its plasma levels both before and after treatment, which might be attributed to active transport or secretion of visfatin from the salivary glands themselves, cellular damage due to edema or cell membrane damage in periodontitis.^32^ Therefore, this biomarker might reflect the functional status of periodontal tissues.

The results of this study also indicated a significant decrease in the clinical parameters of PI, GI, PPD and CAL following non-surgical periodontal treatment, indicating a decrease in the inflammatory load of the disease condition. In addition, it showed a direct relationship between the periodontal tissues status and serum and salivary levels of visfatin, although the relationship was not statistically significant.

Additional long-term prospective studies with larger sample sizes are needed to confirm the results of this study and reveal the exact roll of visfatin in periodontal disease process.

## Conclusion

Based on the results of the preset study, it can be concluded that:

1. Serum and salivary levels of visfatin decreased significantly after non-surgical periodontal treatment.
2. When comparing visfatin concentrations in serum and saliva, there were no significant differences before and after treatment, although the salivary levels were higher.
